# Advances in Extracellular Vesicle Nanotechnology for Precision Theranostics

**DOI:** 10.1002/advs.202204814

**Published:** 2022-11-14

**Authors:** Qian Wu, Siyuan Fu, Hanyang Xiao, Jiaxin Du, Fang Cheng, Shuangshuang Wan, Houjuan Zhu, Dan Li, Fei Peng, Xianguang Ding, Lianhui Wang

**Affiliations:** ^1^ State Key Laboratory of Organic Electronics and Information Displays & Jiangsu Key Laboratory for Biosensors Institute of Advanced Materials (IAM) Nanjing University of Posts and Telecommunications Nanjing 210023 China; ^2^ A*STAR (Agency for Science Technology and Research) Singapore 138634 Singapore; ^3^ Department of Dermatology The Affiliated Drum Tower Hospital of Nanjing University Medical School Nanjing 210008 China; ^4^ Wellman Center for Photomedicine Massachusetts General Hospital Harvard Medical School Charlestown MA 02114 USA

**Keywords:** extracellular vesicle, nanomedicine, nanotechnology, theranostics

## Abstract

Extracellular vesicles (EVs) have increasingly been recognized as important cell surrogates influencing many pathophysiological processes, including cellular homeostasis, cancer progression, neurologic disease, and infectious disease. These behaviors enable EVs broad application prospects for clinical application in disease diagnosis and treatment. Many studies suggest that EVs are superior to conventional synthetic carriers in terms of drug delivery and circulating biomarkers for early disease diagnosis, opening up new frontiers for modern theranostics. Despite these clinical potential, EVs containing diverse cellular components, such as nucleic acids, proteins, and metabolites are highly heterogeneous and small size. The limitation of preparatory, engineering and analytical technologies for EVs poses technical barriers to clinical translation. This article aims at present a critical overview of emerging technologies in EVs field for biomedical applications and challenges involved in their clinic translations. The current methods for isolation and identification of EVs are discussed. Additionally, engineering strategies developed to enhance scalable production and improved cargo loading as well as tumor targeting are presented. The superior clinical potential of EVs, particularly in terms of different cell origins and their application in the next generation of diagnostic and treatment platforms, are clarified.

## Graphical abstract

This review presents recent advances in extracellular vesicle (EVs) nanotechnology for precision theranostic platforms, mainly about EV purification strategies regarding their clinical potential and the recently rapidly developing approach of EV engineering. Their recent theranostic applications are also presented to provide new insights and directions for research and clinical translation of this biomedical vesicles.

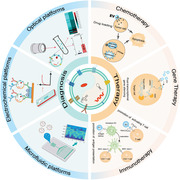

## 1 Introduction

Extracellular vesicles (EVs) are double‐layer lipid membrane vesicles actively secreted by cell.^[^ ^1^ ^]^ After secretion, EVs are released into the extracellular space and enter circulation.^[^ ^2^ ^]^ As a platform of intercellular communication,^[^ ^3^ ^]^ circulating EVs selectively transport biological information from donor cells to recipient cells, including nucleic acids, proteins, and metabolites, which greatly affect the target cells or microenvironment by regulating the expression of gene and proteins.^[^ ^4^, ^5^ ^]^ Hence, EV‐mediated cellular response shows a high impact on many disease processes, such as cancer progression,^[^ ^6^ ^]^ cardiovascular disease,^[^ ^7^, ^8^ ^]^ and neurologic disease.^[^ ^9^ ^]^

Generally, EVs can be classified into three subgroups according to their biogenesis:^[^ ^10^ ^]^ a) Microvesicles/ectosomes/microparticles released from plasma membranes (PM) directly, b) Exosomes that are generated inside multivesicular bodies (MVBs) and released by fusion of internal MVBs with the PM, c) Apoptotic bodies that are secreted by cells undergoing apoptosis. Therefore, EVs are a highly heterogeneous population, reflected in their size, content, and source.^[^ ^11^ ^]^ Cells can shed EVs with different components according to their status.^[^ ^12^, ^13^ ^]^

As a result of the dynamic production of EVs, EVs are valuable biomarkers for diagnosing and monitoring disease.^[^ ^14^ ^]^ EVs have been detected and isolated from various body fluids, such as blood, urine, and even breast milk,^[^ ^15^ ^]^ thereby enabling EV‐based biomarkers to rapid adoption in the clinical arena, i.e., liquid biopsy. Based on this, analytical platforms for detecting EVs have rapidly developed in recent years.^[^ ^16^, ^17^ ^]^ As disease trackers, EVs can offer significant advantages: a) The number of EVs in the biological fluid is at least one order of magnitude higher than that of other circulating biomarkers, such as circulating tumor cells;^[^ ^18^ ^]^ b) Compared with traditional biological protein markers, EVs are more effective because they are secreted by living cells, carrying a great deal of biological information, and are not limited by the fact that circulating tumor DNA (ctDNA) can only detect cells that have died.^[^ ^19^ ^]^ c) The natural lipid bilayer structure of EVs makes their preservation easier and prevents errors in various complex physiological detection scenarios. EVs, however, exhibit unmatched structural advantages when it comes to detection, requiring high sensitivity and specificity, and there are currently no unified standards for this.^[^ ^20^ ^]^ Moreover, a quality control system for EVs has yet to be established. In addition, EV‐based therapy has rapidly developed based on their effect on disease process.^[^ ^21^, ^22^, ^23^ ^]^ Continuing evidence reveals that cellular therapy produces tumorigenicity in vivo.^[^ ^24^, ^25^ ^]^ In contrast, EVs are merely the metabolic state of cells, having no metabolic genetic activity. Therefore, EVs show their safety as a cell‐free therapy. In addition, since EVs have unique biological structures, they are excellent vehicles for delivering drugs: a) Efficient biocompatibility. The “don't eat me” signal on the membrane protect EVs from being phagocytosed by macrophages and reduce their clearance in the circulation;^[^ ^26^ ^]^ b) High penetrability. EVs can cross the blood–brain barrier;^[^ ^27^ ^]^ c) Cell or tissue‐specific targeting due to their natural homing capability.^[^ ^28^ ^]^ In recent years, engineering techniques to modify EV rationally for therapeutic interventions has rapidly developed. Current approaches fall into three categories: a) Gene engineering. Through gene editing technology, EVs could be engineered to display specific guiding peptides and proteins on their membrane that mediate specific biological functions.^[^ ^29^ ^]^ b) Chemical modification. Covalent or noncovalent interactions can be used to introduce a variety of molecules to the EV surface,^[^ ^30^ ^]^ natural and synthetic ligands can also be exposed on their membrane to target specific recipient cell types.^[^ ^31^ ^]^ c) Membrane fusion technique. EVs have a typical biofilm structure. Biofilms are known to have fluidity, so they can be easily fused with other membrane‐containing substances to modify and tune the lipid bilayer membrane.^[^ ^32^ ^]^

**Figure 1:**
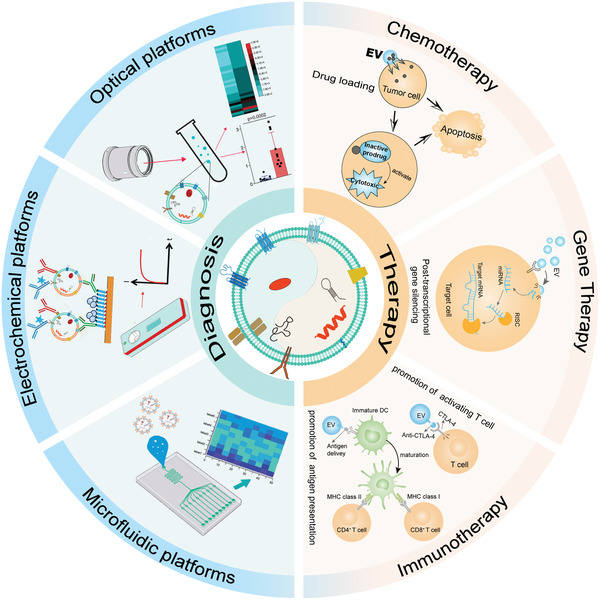
Schematic illustration of EV nanotechnology for precision theranostic platforms. Detection technology for EV feature profiling is constantly updated for disease diagnosis. In this review, optical platform, electrochemical platform, and microfluidic platform in detecting EVs are presented. From traditional chemotherapy, gene therapy, to rapidly developing immunotherapy, EV nanotechnology improves the therapeutic effectiveness for disease treatment.

Herein, we present recent advances in EV nanotechnology for precision theranostic platforms (**Figure** **1**). Notably, EVs exist in a complex body fluid environment and vary considerably in size, morphology, and biochemical components. Thus, improving the purity of EVs is the first step before their application in theranostics. Herein, we discuss conventional and cutting‐edge techniques for EV purification regarding their advantages and limitations. Second, to address the shortcomings of natural EVs in the applications over past years, the recently rapidly developing approach of EV engineering is highlighted in this review. Third, EV detection techniques and recent applications as therapeutic agents are presented to provide new insights and directions for disease diagnosis and treatment.

## 2 EV Purification

A consistent isolation and purification procedure is still one of the major challenges in EV‐based theranostic applications.^[^ ^33^ ^]^ Efficient, simple, and low‐cost EVs purification techniques will help to the investigation of EVs and enable their broader application in areas, such as diagnosis and precise treatment of various diseases. Currently, the most used EVs purification methods include ultracentrifugation, immunoaffinity enrichment, ultrafiltration, and size exclusion chromatography. It is important to note that each of these technologies has its own advantages and inherent limitations that hinder its application. For example, ultracentrifugation shows the advantage of being less expensive and the technology is more mature, but EVs may be damaged by high‐speed centrifugation. Size exclusion chromatography does not have this risk but the method requires the use of specialized equipment. In addition, microfluidic technology offers the advantages of integration and high throughput, but is limited in its capability of treating samples with large quantities. **Table** **1** summarizes the principles, benefits, and drawbacks of these EVs purification methods.

**Table 1:** Current strategies for EV purification

Method	Principle	Advantages	Disadvantages
Ultracentrifugation	Differences in size, density, and shape between EVs and other substances	Low cost; easy to operate; mature	Instrument dependent; long run time; risk of damage to EVs
Immunoaffinity capture	Based on interaction between antigens and antibodies	High specificity; high purity of EVs obtained	High cost; low yield; time‐consuming
Ultrafiltration	Differences in size and molecular weight between particles	No special equipment is required, simple	Easy to clog filter membranes; impurities of similar size
Size exclusion chromatography	Differences in size and molecular weight between particles	Reproducible; inexpensive; simple	Not easy to scale up; requires specialized equipment
Precipitation	protein‐polymer reaction	Easy to operate; high efficiency; without complex devices	Potential contaminants; low purity
Methods based on microfluidics	Microscale purification based on physical properties or biochemical characteristics of EVs.	Cheap; fast; easy to automate	Lack of method validation; Standardization is needed

### 2.1 Ultracentrifugation

Ultracentrifugation is the “gold standard” for EV purification. Statistically, ultracentrifugation method accounts for around 56% of all EV isolation techniques used in EV research.^[^ ^34^ ^]^ Currently, there are mainly two types of ultracentrifugation strategies: differential and density gradient ultracentrifugation.

In differential ultracentrifugation, a lower speed of centrifugation is first used to separate dead cells and cell debris. Then a higher centrifugation speed is employed to separate out the EVs. This method is easy to handle and does not require a high level of expertise from the operator. However, the shortcoming of differential ultracentrifugation is the possibility of damaging EVs during the operation process and the compromised purity in the obtained EVs. Currently, several kits have been developed for differential ultracentrifugation, which provides alternatives to traditional differential ultracentrifugation methods even with limited quantities of biological samples.^[^ ^35^ ^]^

As compared to differential ultracentrifugation, density gradient ultracentrifugation method can yield more pure EVs. By applying a certain centrifugal force to the sample, a density gradient can be created in the centrifuge tube. The components in the sample will settle to the corresponding density zone. The closer the sample is to the bottom, the higher the density. With this density gradient treatment, EVs can be efficiently separated from impurities. The most commonly used density gradient ultracentrifugation method is sucrose density gradient ultracentrifugation. Density gradient ultracentrifugation has some drawbacks for EV separation, such as its long processing time and expensive equipment needed, which limit its usage in the treatment of large‐scale samples.

### 2.2 Immunoaffinity Capture

Immunoaffinity capture is an EV purification technique based on specific antigen‐antibody recognition. Due to the high protein content in the EV membrane, specific immunoaffinity between protein antigen and antibody allows EV isolation.^[^ ^36^ ^]^ Generally, antibodies must be prefixed to a specific particular carrier, which can be magnetic particles or chromatography matrices. Currently, the most commonly used carrier is immunomagnetic beads. Magnetic separation of the EVs from unbound impurities is made possible by antibodies that are modified on beads.^[^ ^37^ ^]^ Compared with differential ultracentrifugation and density gradient ultracentrifugation, immunoaffinity capture shows the advantages of simplicity, specificity and effectiveness. There are, however, limitations to the method, such as antibody quality, cost, etc.

### 2.3 Ultrafiltration

In ultrafiltration, EVs are purified according to their size. By passing samples through different pore sizes of ultrafiltration membranes, EVs can be efficiently purified. Ultrafiltration is particularly suitable for large volume samples, for example, cell cultures and urine.^[^ ^38^ ^]^ The method can be used as a stand‐alone technique or as a complement to ultracentrifugation for separating large microvesicles and EVs.^[^ ^39^ ^]^ In recent years, ultrafiltration has been further combined with other purification methods to improve EV purification efficiency. For example, Xiang et al. proposed a new strategy combining ultrafiltration and TiO_2_ nanoparticles to isolate EVs from human urine. This strategy could not only obtain high‐quality EVs from human urine in 20 min but also yield 91.5% of EVs with intact structure.^[^ ^40^ ^]^ While the ultrafiltration method is simple to handle, it also has some drawbacks, including high protein contamination and easy blockages in the filter pores, which should be addressed in the future EV ultrafiltration technology.

### 2.4 Size Exclusion Chromatography

Size exclusion chromatography is also known as spatial or molecular exclusion chromatography. This method is similar to ultrafiltration, which purifies EVs based on the difference in size. Size exclusion chromatography has many advantages. According to a study that compared the characteristics of urinary EVs obtained by size exclusion chromatography and ultracentrifugation, size exclusion chromatography was superior to ultracentrifugation in terms of recovering particles and proteins, and the purity of EVs extracted with size exclusion chromatography was greater.^[^ ^41^ ^]^ Sidhom et al. concluded that size exclusion chromatography is reproducible, scalable, and low‐cost. Equipment requirements and user expertise are relatively low.^[^ ^39^ ^]^ Based on the above advantages, recent years have seen a dramatic increase in size exclusion chromatography for EV purification.^[^ ^42^ ^]^

### 2.5 Precipitation

As a method of separating viruses and other biological macromolecules, precipitation has been increasingly applied to isolate EVs in recent years. The precipitation purification of EVs involves adding solvents to the sample to alter the solubility or dispersibility of the components. Currently, the reagent commonly used is poly‐Ethylene Glycol (PEG). PEG reduces the solubility of EVs to lead them to precipitate and enables EVs to be obtained by low‐speed centrifugation.^[^ ^37^ ^]^ Coughlan et al. observed that precipitation strategy could be up to 6 times faster than ultracentrifugation, allowing for 2.5 times higher levels of EV concentration in a given volume.^[^ ^43^ ^]^ While this method is simple and does not require much instrumentation, there are still issues such as the tendency to adulterate with impurities and low recovery rates, which may adversely affect the subsequent analysis.

### 2.6 Microfluidics‐Based Methods for EV Purification

As the demand for EV purification grows, new technologies for EV purification are constantly being developed. In addition to the above commonly used EV purification methods, microfluidic technology, with its advantages of integration and automation, offers new ideas for developing EV purification methods with their physical properties or biochemical characteristics.

At present, EVs are mainly purified by size differences in terms of their physical properties. Han et al. developed a size‐dependent microfluidic chip with two symmetrically distributed, serpentine channeled polymethyl methacrylate (PMMA) layers. The PMMA layers have a relatively stable internal structure that improves the stability of the chip. In the middle of the two PMMA layers, there is a nanoporous polycarbonate track‐etched membrane, which ensures efficient cleaning of protein contaminants. To avoid microfluidic blockage, the authors used tangential flow filtration to purify the EVs. The method can provide >97% cleaning efficiency for pollutants and >80% recovery for EVs.^[^ ^44^ ^]^ In addition to taking advantage of the physical properties of EVs, the biochemical characteristics of EVs can also be used to purify EVs combined with microfluidic technology. Tayebi et al. modified streptavidin and biotinylated antibodies on 20 µm of microbeads and then employed antigen‐antibody affinity binding to immobilize and enrich EVs on the surface of the microbeads. Using a microfluidic device equipped with trapping arrays, they were able to efficiently trap microbeads carrying EVs at a single particle level in a microfluidic device.^[^ ^45^ ^]^

Among various methods for purifying EVs, ultracentrifugation, due to its simplicity of operation and mature technology is the most promising strategy for promoting the clinical translation of EVs. In addition, microfluidics‐based EVs purification methods have unique advantages such as high integration and automatable control. The application of microfluidics techniques for EV purification has great potential in clinical testing and diagnosis. However, microfluidics‐based EVs purification methods lack standard and large‐scale experimental tests. Presently, no single EV purification method can be applied to all sample types and application purposes. Different purification methods have their own benefits and disadvantages. Therefore, researchers need to choose the appropriate purification method according to different application purposes. For example, EVs can play an important role in liquid biopsy. There are many impurity particles in blood, so for blood samples, it is more necessary to pay attention to the purity of the EVs obtained by purification. When purifying EVs from blood, size‐exclusion chromatography and immunoaffinity capture can yield EVs of high purity. However, due to the drawbacks of high cost, it is difficult to extend them to a large scale. In terms of treatment, EVs have shown great potential as carriers for drug delivery, however, one of the factors limiting their clinical application is the yield of EVs. Therefore, when purifying EVs, high yield methods such as precipitation can be considered. In addition, although each purification strategies can be used individually, sometimes better results can be obtained by combining different methods. For example, in order to isolate EVs in plasma or cell culture medium, a three‐step combination method of precipitation, density gradient ultracentrifugation, and size exclusion chromatography can be employed, by which EVs with high yield and purity can be obtained.^[^ ^46^ ^]^

## 3 Engineered EVs for Cancer Theranostic Application

Numerous physiological and pathological processes are regulated by the EV‐mediated intercellular communication.^[^ ^47^ ^]^ Therefore, EVs originating from different parent cell lines show great potential for disease treatments.^[^ ^48^ ^]^ However, EV is still a long way away from clinical application, despite several encouraging experimental results. First, although almost all cells can generate EVs,^[^ ^49^ ^]^ the amount of EVs produced is still very limited, which is far from meeting the clinical required amount. In addition, some native EVs cannot perform the desired functions well in vivo and need to be designed for clinical usage in order to make them more usable. Therefore, it is of great significance to improve the yield of EVs production as well as regulate their biological functions to promote clinical translation. This section introduces recent advances in EVs engineering, mainly including genetic engineering, chemical modification, and membrane fusion technology. By applying these strategies, EVs with specific functions can be obtained. **Table** **2** shows the current strategies for EV engineering.

**Table 2:** Current strategies for EV engineering

Methods	Gene engineering	Chemical modification	Membrane fusion technique
Graphic	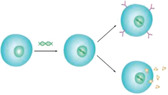	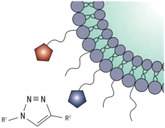	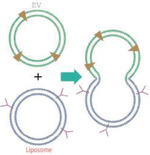
Principle	Target genes are transfected into parent cells or directly loaded into secreted EVs to alter genetic information.	Modification, addition, or removal of various macromolecules, including proteins and nucleic acids, by chemical reactions.	EVs have a phospholipid bilayer membrane structure, and the membrane is mobile and lipophilic.
Strengths	High specificity, improve targeting. When immune cells secrete EVs as parent cells, they themselves can also act as immune factors.	Fast reaction speed, high selectivity, good compatibility. It does not affect the structural integrity of EVs.	Operation is simple, can solve the problem of EVs in vivo easy degradation, short half‐life and so on, improve the bioavailability of EVs, and can give them targeting.
Limitations	Most of the transfection reagents have certain toxic and side effects on cells. Its stability and safety need to be studied.	The complexity of EVs surface may reduce the reaction efficiency and even endanger the structure and function of the vector.	Low specificity Low reaction efficiency May destroy the structural integrity of EVs.

### 3.1 Gene Engineering

With genetic engineering, certain nucleic acids can be feasibly loaded into donor cells and then transferred to secreted EVs.^[^ ^50^ ^]^ The nucleic acids can also be directly introduced to EVs by electroporation approach. Using these strategies, EVs can be designed according to requirements. Through genetically modifying and designing EVs: a) The yield of EVs can be enhanced; b) Drug delivery efficiency and targeting capability of EVs can be improved; c) The multifunctionality and biosafety of EVs for cancer and other disease treatment can be strengthened.^[^ ^51^ ^]^

**Figure 2:**
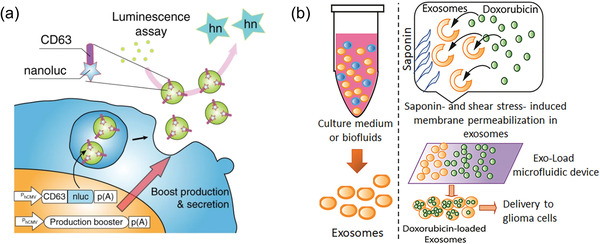
Gene engineering. a) Schematic illustration of gene engineering boost EVs production. Reproduced with permission.^[^ ^52^ ^]^ Copyright 2018, Springer Nature. b) Schematic illustration of drug loading into EVs using the Exo‐Load microfluidic technology. Reproduced with permission.^[^ ^59^ ^]^ Copyright 2020, DOVE Medical Press.

Genetic engineering can be manipulated to enhance EV secretion from donator cells. To boost mass EV generation from donor cells, developed an exosomal transfer device was designed by Kojima et al. (**Figure** **2**a).^[^ ^52^ ^]^ In their device, different plasmids were designed to enhance the packaging of RNA and to assist the secretion of endocellular EVs. As a result of plasmid introduction, they found a 40‐fold increase in EV production, at the same time without noticeable size and distribution change. In addition, using Lamp2a fusion protein, Sutaria et al. inserted the gene encoding pre‐miR‐199a into an artificial intron.^[^ ^53^ ^]^ In EVs, the miR‐199a was enriched up to 65‐fold. Although the amount of miRNA loading to EVs was relatively low by this method, the loading strategy would be applicable to other drug delivery methods, such as miRNA mimics and RNA with hairpin sequences.

It is also possible to improve the targeting capability of EVs through genetic engineering in addition to increasing the production of EVs. By transfecting Expi293F cells to express AntiCD3 (CD3 antibody), antiepidermal growth factor receptor (anti‐EGFR), and platelet‐derived growth factor receptor fusion protein on the EVs membrane,^[^ ^54^ ^]^ Cheng et al. constructed engineered EVs with high affinity to both breast cancer cells and T cells. These engineered EVs can specifically enrich T cells to the vicinity of breast cancer cells, therefore improving the targeting ability of activated T cells to breast cancer cells.

Besides, postsecretion loading is another genetic engineering strategy that can directly process EVs to load therapeutic molecules such as RNA. Compared to presecretion loading, postsecretion loading is relatively easy to use and has been widely in recent years. There are several postsecretion loading methods available, including electroporation, extrusion, and freeze/thaw cycling. For loading RNA into EVs, coincubation,^[^ ^55^ ^]^ electroporation,^[^ ^56^ ^]^ and sonication^[^ ^57^ ^]^ are classic methods.^[^ ^58^ ^]^ Additionally, Thakur et al. reported using a microfluidic device called Exoload to load EVs with shear stress (Figure 2b).^[^ ^59^ ^]^ Alternatively, O'Loughlin et al. utilized the lipophilic structure of cholesterol for gene engineering.^[^ ^60^ ^]^ In their research, they demonstrated that siRNAs conjugated with triethylene glycol and cholesterol were highly effective in loading into EVs. By varying the incubation time, volume, temperature, and EVs/siRNA ratio, they successfully loaded EVs with cholesterol‐conjugated siRNA. Furthermore, proteins capable of binding both nucleic acids and EVs have also been reported to load nucleic acids into EVs.^[^ ^61^ ^]^

### 3.2 Chemical Modification

**Figure 3:**
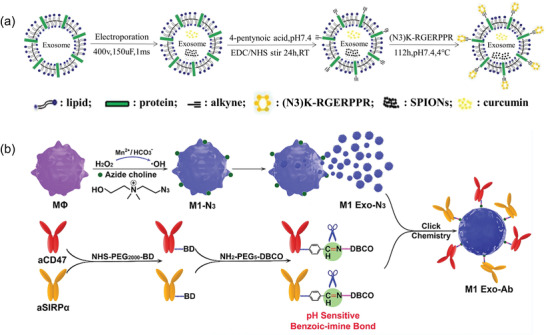
Chemical modification strategies for EV. a) Schematic representation of RGE‐Exo‐SPION/Cur synthesis. Reproduced with permission.^[^ ^63^ ^]^ Copyright 2018, Elsevier. b) Schematic illustration of Mn^2+^ induced M1 polarization of macrophages and the design of M1 Exo modified with aCD47 and aSIRP*α*. Reproduced with permission.^[^ ^65^ ^]^ Copyright 2020, Wiley. RGE: neuropilin‐1‐targeted peptide (RGERPPR); SPIONs: superparamagnetic iron oxide nanoparticles; Cur: curcumin.

Chemical modifications can be divided into covalent and noncovalent methods, in which covalent modifications bind through covalent bonds, and in addition, unlike cells, EVs are nonbiological entities, so biological affixation and “click chemistry” reactions can be introduced to modify EVs membranes without damaging their biological activity.^[^ ^62^ ^]^ Noncovalent modifications are to bind specific substances to EVs' membranes through noncovalent bonds, including electrostatic interactions and classical interactions, etc. Noncovalent binding requires relatively mild reaction conditions for binding, and compared with covalent modifications, the bond energy is smaller and the binding strength is weaker. Unlike genetic engineering, chemical modification involves modifying the surface of EVs, rather than the core of the particle. Chemical modifications directly conjugate ligands to EVs surface through chemical reaction. For example, EV surface protein has numerous amino groups that can be used as ligation targets. In an azide‐alkyne cycloaddition “click” reaction, proteins on the membranes of the EVs can be converted from amines to alkynes. Chemical conjugation offers the advantage of high selectivity and compatibility without affecting the structural integrity of EVs. In a click chemistry approach, Jia et al. conjugated neuropilin‐1 to the surface membrane of EVs (**Figure** **3**a).^[^ ^63^ ^]^ Following that, superparamagnetic iron oxide nanoparticle and curcumin were loaded into the EVs for imaging‐guided glioma therapy. Through a copper‐free azide‐alkyne cycloaddition reaction, Tian et al. demonstrated bio‐orthogonal conjugation of the cyclic (arginine‐glycine‐aspartate‐*d*‐tyrosine‐lysine) peptide [c(RGDyK)] to the surfaces of EVs.^[^ ^64^ ^]^ As a result of the EV conjugation, a high affinity and selectivity was demonstrated in the treatment of cerebral ischemia. Additionally, Nie et al. used a pH‐sensitive linker to combine azide‐modified EVs with dibenzocyclooctyne‐modified antibodies of CD47 and signal regulatory protein alpha (Figure 3b).^[^ ^65^ ^]^ When injected without modification, most EVs are preferred to retain in liver or spleen rather than in the desired tumor sites.^[^ ^66^ ^]^ Through covalently conjugating EVs with cyclic RGD peptide, enhanced antitumor effects can be improved by targeting tumor cells.^[^ ^67^ ^]^ In addition, noncovalent strategies have also been leveraged to provide stable modification on EV membranes. Amstrong et al.^[^ ^68^ ^]^ exploited electrostatic interactions to bind positively charged lipids to the EVs surface, thereby enhancing EVs uptake. Similarly, Qi et al. successfully bound blood EVs with transferrin‐labeled superparamagnetic nanoparticles to target the native transferrin receptor present on EVs membrane.^[^ ^69^ ^]^

### 3.3 Membrane Fusion Technique

With phospholipid bilayer membrane structure, EVs can readily be fused with other types of exogenous lipid membrane structures by freeze‐thaw cycles, filtration or extrusion methods.^[^ ^70^ ^]^ In comparison to other strategies for EVs modification, membrane fusion is easy to operate, which can be applied to significantly improve the bioavailability of EVs by preventing the degradation of EVs in vivo as well as improving their half‐life. Additionally, membrane fusion can facilitate drug delivery, especially for those that are poorly loaded in EVs without changing the chemical structures.^[^ ^71^ ^]^

Applying the freeze‐thaw method, Sato et al. developed a hybrid exocapsule by fusing vesicle membrane with functionalized liposomes.^[^ ^72^ ^]^ With liposomes embedded with peptides or antibodies as donators, EVs surface can be readily modified with target moieties after the membrane fusion strategy. Chakravarti et al. prepared an artificial extracellular vesicle using the membrane fusion method.^[^ ^73^ ^]^ They passed human adipocyte stem cells through a series of filters and continuous centrifugation to remove the intracellular contents. Finally, the dividing mother membrane spontaneously formed extracellular vesicles. The EVs produced by this method exhibited excellent stability over three weeks and demonstrated a strong ability to target drug delivery without any significant cytotoxicity.

**Figure 4:**
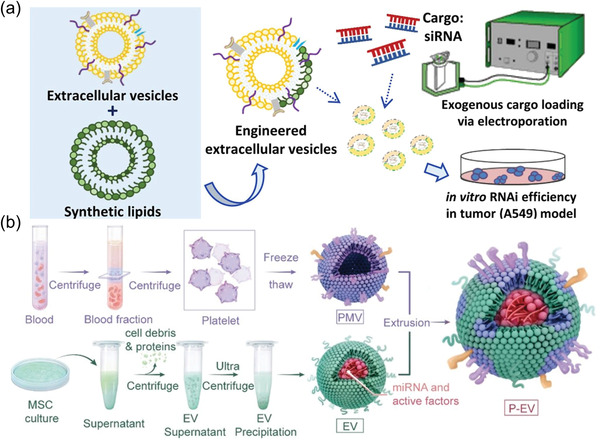
Membrane fusion technique. a) Engineered EVs obtained by extrusion serve as new drug delivery platforms. Reproduced with permission.^[^ ^74^ ^]^ Copyright 2020, Elsevier. b) Schematic diagram of P‐EVs preparation. Reproduced with permission.^[^ ^77^ ^]^ Copyright 2021, Ivyspring International.

The insufficient EV secretion poses a major challenge for EV‐based clinic translation. To enable large‐scale of EV production, Jhan et al. mass‐produced engineered EVs by combing EVs with lipid‐based materials. Particle number analysis showed that the extrusion method allowed 6‐ to 43‐fold of vesicle generation (**Figure** **4**a).^[^ ^74^ ^]^ Their results shown that the surface composition and function of EVs can be modulated with lipid extrusion, allowing for mass‐produced while maintaining their targeting capability. In fact, lipids can also be leveraged to directly label cell membranes to form EVs‐mimicking vesicles.^[^ ^75^ ^]^ Wan et al. conjugated nucleolin targeting aptamer AS1411 with Cholesterol poly (ethylene glycol).^[^ ^76^ ^]^ The conjugate was immobilized on the membrane of mouse dendritic cells. By microshrinkage extrusion, cells were passed through two filters with pore sizes of 10 and 5 µm to generate EVs‐mimicking vesicles. Using ultrasound, paclitaxel can be loaded in EV and delivered in vivo to fight cancer. These results suggested extrusion cells are a fast, simple, and economical way of preparing sufficient drug delivery vehicles containing ligands. In addition, due to the lack of targeting ability of natural EVs, Li et al. utilized membrane fusion method to construct platelet‐engineered EVs (P‐EVs) for therapeutic angiogenesis. Taking advantage of platelets' natural endothelium tropism and the proangiogenic capability of EVs originated from bone marrow mesenchymal stem cells, hybridization with two different membranes could be achieved through direct membrane fusion (Figure 4b).^[^ ^77^ ^]^ The obtained EVs exhibited both angiogenesis and targeting capability.

### 3.4 EV Engineering for Drug Loading

EV engineering strategies are meaningful for drug delivery. Recent years, the application of EVs as drug carriers has attracted great interest due to their high biocompatibility, low immunogenicity, circulating stability, and easy of crossing physiological barriers. Compared with traditional drug loading methods such as coincubation or electroporation, EV engineering can expand drug loading types and improve efficiency considerably. For example, the genetic engineering of EVs is capable of loading various nucleic acid drugs ranging from small ligands such as miRNA or siRNA to DNA structures like plasmids. Generally, genetic engineering for drug loading can be classified as presecretion drug loading and postsecretion drug loading. The former are mostly loaded into donor cells using a gene transfection method, such as transfection of miRNA‐let7c into mesenchymal stem cells.^[^ ^78^ ^]^ The latter involves loading drugs directly into EVs, then using their natural targeting properties to deliver nucleic acid‐based therapeutics directly to target cells.^[^ ^79^ ^]^ Due to the abundance of protein on EV membranes, drugs that are difficult to enter can also adopt chemical modification strategies to attach on the membrane of EVs. For example, targeted RGDyK ligands can be coupled to EVs surface by click chemistry.^[^ ^64^ ^]^ Often, it is difficult for drugs to be introduced into the EVs themselves but must be present inside the EVs to be effective. In order to make these drugs effective, membrane fusion strategies can be employed by fusing EVs with drug‐loaded liposomes to alter the natural membrane structure of EVs so that the drugs can enter into EVs. It has been reported that doxorubicin can be loaded into hybrid EVs by the above membrane fusion strategies.^[^ ^80^ ^]^ In summary, due to the different physicochemical properties of small molecule drugs or biological macromolecules, appropriate EV engineering strategies should be considered to ensure their drug‐loading efficiency.

## 4 Detection

The current research shows that the detection and analysis of EVs can be divided into two levels: characterization and content.^[^ ^49^, ^81^ ^]^ EVs participate in human physiological activities in the form of physiological signals. This bioactive molecule participates in processes, such as cell proliferation, inflammation, tumor immunity, and metastasis. Because disease‐related proteins and genetic materials are often loaded into EVs,^[^ ^82^ ^]^ especially in different tumor cells, there are specific differential expressions. This has broad prospects for revealing the principles of diseases, especially immune diseases.^[^ ^83^ ^]^ First of all, it is manifested in the early diagnosis of diseases, and there can be a more intuitive quantitative standard for the development of diseases.^[^ ^84^ ^]^ However, EVs have only a very small content at the microlevel of biological cells, there are strict requirements for the efficiency of extraction procedures and the sensitivity of detection methods. For the detection of EVs from a small number of samples, at present, most of them are optimized traditional monitoring and analysis tools, which are greatly limited in clinical practicality, especially in sensitivity and analysis volume. With a focus on EV contents, here we summarize the technical direction and platforms in recent years for EV detection, which realized the engineering application of the new EVs detection technology by combining different technology platforms.

### 4.1 EVs Characterization and EVs Contents

#### 4.1.1 Morphological and Quantitative Characterization

The morphological characterization of EVs is an indispensable part of the identification and detection of exosomes. For the morphological characteristics of EVs, traditional laboratory methods include scanning electron microscope (SEM),^[^ ^85^ ^]^ perspective electron microscope (TEM),^[^ ^86^ ^]^ atomic force microscope (AFM),^[^ ^87^ ^]^ and optical reconstruction microscope.^[^ ^88^ ^]^ SEM can collect the electrons scattered from the target sample for imaging, TEM has the ability to display the internal structure of EVs and AFM can form 3D images through multiple parameters. It is worth noting that among the three methods, except for atomic force microscopy, dehydrated, or fluorescent staining needs to be pretreated before detection, which affects the activity and morphology of the sample. Tian et al. chose a cryo‐electron microscopy (cryo‐EM),^[^ ^89^ ^]^ which can avoid the fixed dehydration of the samples and reduce the damage to the samples. In general, morphological characterization is not conducive to the detection of a large number of samples.

In order to further improve the accuracy of the detection of exosomes, the quantitative characterization of exosomes has mainly used optical means for auxiliary analysis. Dynamic light scattering (DLS)^[^ ^90^ ^]^ and Nanoparticle tracking analysis (NTA)^[^ ^91^ ^]^ are widely used to determine the real‐time concentration and size of particles. The former usually uses the intensity of scattered light interference to accurately detect uniform medium samples, but it has poor effect on nonuniform media, such as protein aggregates. NTA is a method to collect the light signal scattered by the sample after being irradiated by the light beam using an optical microscope.

#### 4.1.2 EVs Contents

**Figure 5:**
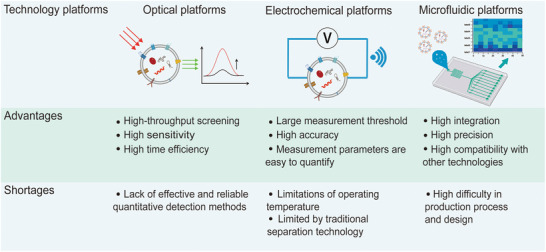
Advantages and limitations of various detection platforms.

Numerous studies have suggested that EVs contents are associated with cancer generation and metastasis. Therefore, it is suggested that the detection of EV content is valuable for cancer diagnosis. While Western blot and ELISA (enzyme‐linked immunosorbent assay) are two commonly adopted biological detection approaches,^[^ ^92^ ^]^ both of these methods have certain limitations in detection sensitivity.^[^ ^93^ ^]^ In recent years, with a deeper understanding of EVs, a large number of technical means for EV detection have been developed based on electrochemical and optical platforms.^[^ ^94^ ^]^ In addition to this, microfluidic systems are increasingly developed for the detection of EV contents. These integrated platforms have unique advantages in terms of sensitivity, specificity, and convenience (**Figure** **5**). The modalities and their potential applications in EV biosensing have been discussed in the following sections.

### 4.2 Optical Methods for EV Detection

Optical technologies show good precision in measuring biological targets in vitro and in vivo. Similar to the optical means of quantitative EV characterization, optical technology is well‐suited for large‐scale screening operations. For the detection of circulating biomarkers like EVs, optical means offer high sensitivity, and timeliness of detection.^[^ ^95^ ^]^ Many optical approaches, such as fluorescence, surface plasmon resonance (SPR), colorimetric tests, and Raman scattering, have been developed for EV detection. In this section, we summarize the recent advances in optical strategies for EV detection.

#### 4.2.1 Fluorescence Detection

**Figure 6:**
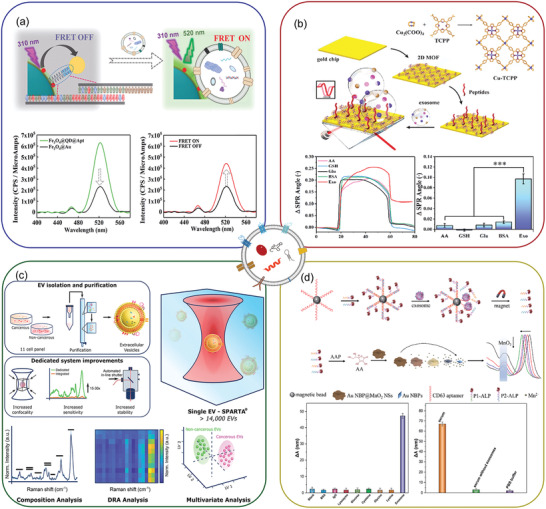
Optical methods for EV detection. a) Fluorescence detection. Reproduced with permission.^[^ ^99^ ^]^ Copyright 2021, Royal Society of Chemistry. b) Surface plasmon resonance detection. Reproduced with permission.^[^ ^103^ ^]^ Copyright 2022, Elsevier. c) Surface‐enhanced Raman scattering. Reproduced with permission.^[^ ^106^ ^]^ Copyright 2021, American Chemical Society. d) Colorimetric detection. Reproduced with permission.^[^ ^108^ ^]^ Copyright 2020, American Chemical Society.

Aiming at high sensitivity and standardized standards, fluorescence method is increasingly developed in laboratories for EV detection. For complex clinical samples, it is a problem to solve the high background signal so as to obtain more sensitivity and specificity EV signal. Besides, the clinical environment does not usually have the same professional resources and space‐time characteristics as laboratories. For the clinical setting, it is desirable to develop more reliable and more accurate fluorescence detection methods that allow point‐of‐care testing.^[^ ^96^ ^]^ Generally, there are mainly two types of fluorescence detection: direct recognition and indirect recognition. Direct recognition involves the binding of fluorescent aptamers or antibodies with antigens expressed on the membrane of the EVs. Based on this mechanism, Li et al. designed a bionanofluorescent sensor utilizing homogeneous magnetic‐fluorescent exosomes, during the operation of this sensor, tumor‐derived EVs were immunomagnetically captured, and the aggregation‐induced emission luminogens/graphene oxide (AIEgen/GO) sensor was matched with a DNA amplification method, and the detection limit could reach 6.56 × 10^4^ particles µL^−1^ within 2 h.^[^ ^97^ ^]^ Yu et al. also used the detection method based on CD63 aptamer, which can be combined with the aptamer modified on the surface of magnetic beads. When EVs were captured, the CD63 labeled EVs lead to the short sequence in the supernatant fall off, and then the concentration of EVs was quantitatively detected by the fluorescence intensity in the supernatant.^[^ ^98^ ^]^ Focusing on nonradiative fluorescence resonance energy transfer (FRET), Zhu et al. designed a magnetic aptamer sensor based on this phenomenon, enriched EVs by the magnetic attraction of aptamers, and detected lung cancer cell proteins using the FRET phenomenon (**Figure** **6**a). The system showed super sensitivity with the minimum detection threshold reaches to 13 particles mL^−1^.^[^ ^99^ ^]^ Meanwhile, it has the ability of FRET to quench fluorescence when graphene oxide was combined with fluorescent dyes. It is worth mentioning that this fluorescence detection strategy belongs to the indirect mode. Fluorescence biosensors often judge the detection results by the “on‐off” change of the light signal, which can greatly improve the signal‐to‐noise ratio in fluorescence signal detection. For example, when adsorbed on a graphene oxide film, the fluorescence of FAM‐labeled aptamer is quenched, while the target EVs compete with the aptamer and redisplay the fluorescence signal.^[^ ^100^ ^]^ These optical platforms after engineering transformation greatly improved the target signal strength, and possess a good improvement in fluorescence detection performance.

#### 4.2.2 Surface Plasmon Resonance Detection

Compared to fluorescence‐based methods, surface plasmon resonance (SPR) allows for better monitoring of biomolecular binding without additional labeling.^[^ ^101^ ^]^ SPR techniques are based on measuring the optical change in the refractive index caused by the adsorption of sample molecules at the film/liquid interface. The detection distance of gold surface binding events by SPR is less than 200 nm, which matches the size of EVs. Therefore, SPR‐based biosensors are very attractive for the study of EVs. In recent years, SPR has been increasingly adopted for various EV detection. With the assistance of dual gold nanoparticles (AuNPs), Wang et al. designed a sensitive SPR sensor. The amplification of the EV signal was achieved through electronic coupling between Au film and Au particles and the controlled hybrid connection of AuNPs generated by the coupling effect in the plasma nanostructure. The method is highly sensitive, with a limit of detection (LOD) of 5 × 10^3^ EVs mL^−1^.^[^ ^102^ ^]^ However, the disordered distribution of nanoparticles can interfere with the optical response of surface plasmon resonance, which limits its practical application. 2D materials have high electron mobility and light absorption. With no interference with biomolecular interactions, 2D materials can enhance SPR signals, which can improve the sensitivity of SPR and provide accurate measurements of target markers. Therefore, for stably SPR sensors, it is necessary to find a 2D material with a highly ordered internal structure, good optical performance, and good dispersion. To develop a fast and highly sensitive SPR sensor, Wang et al. developed a metal organic framework (MOF)‐based SPR sensor (Figure 6b). They deposited the synthesized 2D MOF directly on the surface of the gold chip by a simple hydrothermal method and found a larger specific surface area, hence the sensor possesses more active sites to bind with biomolecules and obtained more concrete biological information.^[^ ^103^ ^]^ Moreover, SPR technology does not require additional labels, allowing real‐time measurement without damage to samples.

#### 4.2.3 Surface‐Enhanced Raman Scattering

Optical biosensors based on nanomaterials have been widely employed for accurate EV detection. Based on SPR, surface‐enhanced Raman spectroscopy (SERS) has become an excellent optical analysis technology in EV. The signal of molecular attached on specific metal surface can be amplified by both electromagnetic and chemical mechanisms.^[^ ^104^ ^]^ Overall, It is used in the field of mobile medicine due to its potential to significantly enhance the sensitivity at the single molecule level. Pang et al. designed a rapid indirect SERS analysis to detect EV PD‐L1 with PD‐L1 antibody‐modified Au@Ag probes. Their results show that the SERS strategy can complete the detection within an acceptable timeframe.^[^ ^105^ ^]^ Raman spectroscopy has high efficiency and marker signal enhancement advantages and can realize nondestructive detection of EVs. The relevant data collection can be automated, which is a good improvement for quantifying detection data. Jelle et al. developed an automated Raman spectral analysis system based on a single‐particle analysis. Using a reduced dimension visualization system, they collected the spectral data of more than 14 000 EVs and distinguished cancer from noncancer cells (Figure 6c).^[^ ^106^ ^]^ Therefore, Raman spectroscopy offers exciting potential for EV detection and analysis.

#### 4.2.4 Colorimetric Detection

Colorimetry is another optical detection technique for EV detection based on chroma biosensors. The colorimetric analysis allows the concentration of chromogenic samples to be observed with the naked eye, allowing rich serum samples to be processed and to display “yes/no” answers or semiquantitative results without additional analytical instruments.^[^ ^107^ ^]^ However, the difficulty of visual observation is the uncontrollable factor of sensitivity. EV detection based on colorimetry should start by amplifying the signal intensity and improving the colorimetric matrix. Zhang et al. designed a highly sensitive colorimetric biosensor combined with local surface plasmon resonance (LSPR) to quantify EVs, and the EVs started a competitive reaction. In this process, the amount of alkaline phosphatases (ALPS) is measured directly by generating alkaline phosphatases, which effectively enhances the target signal. Second, according to LSPR, AuNBP@MnO_2_ NS was selected. Its combination of plasma and optical detection greatly improves sensitivity, meanwhile, benefiting from the signal amplification of competitive reaction and superior refractive index sensitivity of colorimetric substrate, the final detection limit can reach 1.35 × 10^2^ particles µL^−1^ (Figure 6d).^[^ ^108^ ^]^ The results of this study indicate that the traditional colorimetric method does not have superiority over contemporary research methods and that the colorimetric sensor designed by plasma exhibits a greater sensitivity and difference, which indicates that the application of plasma‐based colorimetric methods in the future will be extremely beneficial.

#### 4.2.5 Comparison of Different Optical Platforms

According to the previous classification, the above four EV detection methods belonging to optical means all show excellent accuracy and sensitivity in measuring biological targets. Coincidentally, fluorescence detection and colorimetric detection are mainly divided into two categories: paper‐based sensors and solution‐based sensors. Paper‐based sensors usually use paper as a substrate, which is beneficial for instant detection due to its simple and convenient use and small sample size. Solution‐based fluorescent or colorimetric sensors typically rely on enzymes, fluorescent nanoparticles, or engineered signaling tags to induce signal changes. In the previous period, there were some challenges in quantifying fluorescent signals in a nonlaboratory environment. Li et al. have developed a high‐affinity recognition and visual extracellular vesicle test (HARVEST). When EVs are captured by nanomaterials, the fluorescence signal can be induced after chemical labeling. With the help of smartphones, the quantitative analysis of the number of EVs can be quickly carried out, which helps to enrich the EV fluorescence detection methods in mobile scenes.^[^ ^109^ ^]^ Both SPR and SERS have advantages of the high sensitivity and label‐free real‐time detection. In particular, although label‐free SERS assay is a simple method to identify biomolecules, it has limitations in clinical application. Many important oncogenic EVs exist in biofluids with low enrichment, and the probability of target EVs matching with SERS substrates may be reduced due to the nonspecific‐adsorbed of other molecules, while the SERS signal of coadsorbed molecules, making the data analysis more complex. Therefore, compared with the other three detection methods, SERS labeled with antibodies or aptamers also has unique spectral signals to recognize in complex biological environments, especially when using more than one SERS tags, multiple biomarkers in a sample can be detected in a cycle to meet the requirements of accuracy and efficiency.

### 4.3 Electrochemical Methods for EV Detection

**Figure 7:**
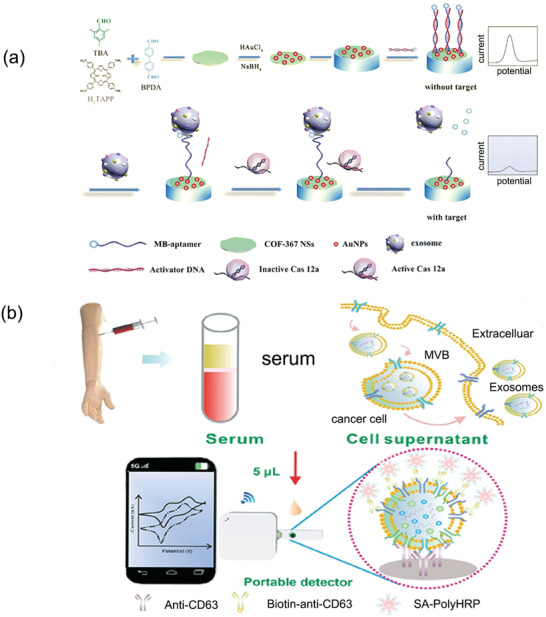
Examples of EV detection using electrochemical sensors a) Schematic illustration for designing the electrochemical biosensor for PD‐L1^+^ EV detection. Reproduced with permission.^[^ ^104^ ^]^ Copyright 2022, Elsevier. b) A scheme of electrochemical biosensors combined with smartphones for direct detection of EVs derived from serum. Reproduced with permission.^[^ ^112^ ^]^ Copyright 2022, American Chemical Society.

The electrochemical measurement method is used to accurately measure the electrochemical potential or current of the sample, which has the advantages of a large measurement threshold and good accuracy.^[^ ^110^ ^]^ Typically, a designed specific aptamer or antibody is first employed to bind EVs, and bioelectric signals are generated in the binding process, which was used for the quantitative analysis of EVs. using a signal amplification strategy of 2D hierarchical nanosheets (2D COF NS_S_) and AuNPs to defunctionalize 2D skeleton nanosheets, Wang et al. reported an electrochemical method to detect PD‐L1^+^ EVs. They found this hybrid strategy can improve the detection range to 1.2 × 10^7^particles µL^−1^ (**Figure** **7**a).^[^ ^111^ ^]^ The choice of key biorecognition elements for electrochemical sensors depends entirely on the binding affinity and efficiency between the recognition element and the target. In this regard, the most classic membrane protein recognition element is the “lock and key” model of antigen‐antibody interaction. Su et al. developed an EV biosensor that can measure prostate cancer and display data on smartphones (Figure 7b). They created a multienzyme signal gain mechanism based on the double‐antibody sandwich method to detect exosomes. CD63‐expressing EVs associated with prostate cancer could be detected within a short time. The measured parameters after the final conversion showed good strong correlation characteristics. This EV sensing platform added a new rapid means of detection.^[^ ^112^ ^]^ In addition, as in the above study, CD63 was used as a protein marker for EVs detection, but it should be noted that since normal EVs and disease‐related EVs both contain a large amount of CD63, it may lead to the amplification of background noise. Therefore, it is more desirable to utilize cancer‐related specific markers. As in the example below, Sun et al. proposed an efficient and label free electrochemical biosensor, whose backbone is mainly composed of Zr‐based metal‐organic frameworks (Zr MOFs), which can be used to detect secretions derived from glioblastoma (GBM). In principle, peptide ligands can interact with human epidermal growth factor receptor (EGFR) overexpressed in GBM derived exosomes. The modified Zr‐MOFs can be adsorbed on the surface of EVs, and then the concentration of EVs can be determined by quantifying the electroactive molecules on the MOF. The final performance range is 9.5 × 10^3^ to 1.9 × 10^7^ particles µL^−1^ and the limit of detection is 7.83 × 10^3^ particles µL^−1^, confirming the prospect of early diagnosis of GBM and other potential diseases.^[^ ^113^ ^]^

Electrochemical methods have natural advantages in clinical scenes and mobile workspaces compared to optical methods. They can be effectively integrated with other separation techniques. Combining electrical signals with high‐performance chips makes it possible to evaluate the detection effect in real‐time and further enhance the efficiency of EV detection.

### 4.4 Microfluidic Platform for EV Detection

In conventional EV capture and detection, a large number of samples are needed and multistep are involved. In contrast, microfluidic methods can process samples at a microscale with multiplexed advantage. They can integrate almost all processing units, such as EV capture and detection parts into one platform.^[^ ^114^ ^]^ Compared with traditional detection methods, the most significant advantage of microfluidic detection platform is that it can increase the enrichment of EVs without a heavy overspeed separation process. As a result of targeted design, microfluidic systems can increase the chance of binding between EVs and specific antibodies, thereby increasing the possibility of detecting EVs in a timely manner, and this could potentially lead to a rapid EV detection technique using microfluidic system.^[^ ^115^ ^]^

**Figure 8:**
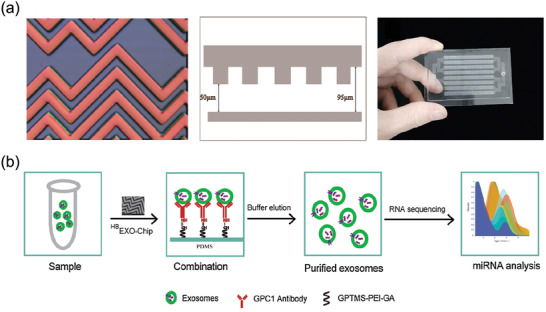
An example of microfluidic platform for EV detection. a) The herringbone mixer structure of ^HB^EXO‐chip at various angles. b) Scheme of exosomes capture and release. Reproduced with permission.^[^ ^117^ ^]^ Copyright 2021, Elsevier.

The most used microfluidic method for EV detection is based on immune affinity recognition.^[^ ^116^ ^]^ Based on immune affinity, Zhang et al. developed a microfluidic device ^HB^EXO‐chip (**Figure** **8**a). The device can use tumor target (Glypican‐1) to capture EVs, and the error can be effectively reduced by specific protein markers. The marker gpc1^+^ used in the study can effectively distinguish pancreatic cancer from the control group, providing a feasible and effective way to detect pancreatic cancer (Figure 8b).^[^ ^117^ ^]^ With a shuttle flow mode of the microfluidic method, Chen et al. could separate the required EVs in a quarter of an hour without labels. miRNA sequencing and real‐time PCR were performed on EV isolated from breast cancer cells. With this strategy, they identified a strong correlation between EVs hsa‐mir‐18a‐3p and the metastasis of breast cancer cells.^[^ ^118^ ^]^

The extensive involvement of the microfluidic platform in the biological field provides a very suitable environment for EV detection. All the steps of EV detection procedure can be handled on the same platform, which saves time and money. This intensive model provides the possibility of industrialization and mass production for the clinical application of detecting EV and provides an effective path for EV to go out of the laboratory and realize practical application.

## 5 Engineered EVs in Disease Treatment

EVs are involved in immune regulation and transfer of genetic material, highlighting their role in cell–cell communication. The effect of EVs on recipient cells varies depending on their derived cells. For instance, EVs derived from Mesenchymal stem cells (MSCs) have the ability to induce angiogenic programs in delivering immunomodulatory signals and quiescent endothelial cells. In contrast, EVs originated from immune cells have been confirmed to contain major histocompatibility complex (MHC),^[^ ^119^ ^]^ showing the ability to induce or suppress specific immune responses. Therefore, the cell derivation of EVs should be taken into account first when utilizing them as therapeutic agents. Besides, due to their natural source, EV has efficient biocompatibility and biodegradability, enabling them to avoid phagocytosis with reduced immunogenicity and prolonging half‐life in vivo. These advantages hold great promise for the use of EVs as drug delivery vehicles. There are, however, several issues with the use of natural EVs for disease treatment that need to be addressed. For example, EVs are prone to being trapped and stuck in nonspecific tissues, particularly the liver and lung, which results in insufficient targeting of EVs.^[^ ^120^ ^]^ Several pieces of evidence suggest that the engineering of EVs can significantly improve their performance, such as targeting ability and drug loading capacity. Thus, the latest advances in engineered EVs for disease treatment will be focused on in this part.

### 5.1 EV Derived from Different Sources

Depending on cell origins, EVs display a set of cell‐specific proteins, which determine their specific functions and fate.^[^ ^10^ ^]^ Therefore, the cellular source should be taken into consideration in order to maximize therapeutic effect. In this part, we discuss EVs derived from MSCs, tumor cells, macrophages and platelets in the application for disease treatment.

#### 5.1.1 MSC‐Derived EVs

The multidimensional differentiation potential of MSCs, as well as their hematopoietic support, immune regulation, and self‐replication properties, have drawn the attention of researchers continuously.^[^ ^121^, ^122^, ^123^, ^124^ ^]^ Aside from differentiating, adult stem cells also release paracrine substances that contribute to various tissue repair. EVs are included in the secretome of stem cells, which target a number of biological pathways through paracrine effects.^[^ ^125^ ^]^ The paracrine hypothesis provides evidence for using MSC‐derived EVs as a cell‐free therapy alternative to MSCs. Here, we focus on the repair capability of MSC‐derived EV and summarize their latest applications in regenerative medicine.

##### Myocardial Ischemia/Reperfusion Injury (MI/RI)

**Figure 9:**
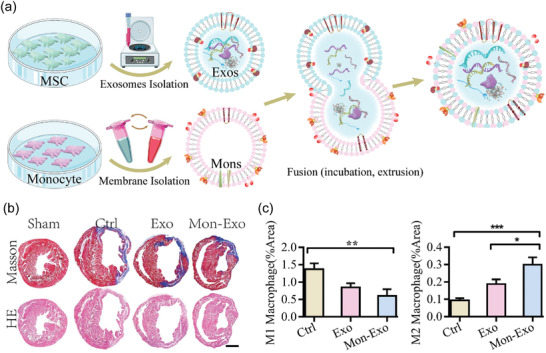
The therapeutic effect of MSC‐derived EVs on MI/RI. a) Schematic diagram of the construction of EV‐Monocyte mimics (Mon‐Exos). b) Heart sections were taken from a MI/RI model mouse after 4 weeks of treatment. Masson and HE staining were performed to examine collagen volumes and infarct areas. c) Mon‐Exos significantly promoted M2 polarization while reducing the number of M1 cells, regulating macrophage subpopulation homeostasis. Reproduced with permission.^[^ ^127^ ^]^ Copyright 2020, Elsevier.

The therapeutic effect of MSC on MI/RI is mainly through reducing tissue damage or enhancing tissue repair. As early as 2009, Lai et al. reported that purified MSC‐secreted EVs could significantly reduce cardiac infarct size in vivo, demonstrating the potential of EVs in heart regeneration and repair postinfarction.^[^ ^126^ ^]^ However, Natural EVs are still hindered by poor homing efficiency, which limits their clinical application. As a means of improving the efficiency of EV delivery to the injured myocardium, Zhang et al. created EV‐monocyte mimics (Mon‐Exos) through the use of membrane fusion technologies (**Figure** **9**a). Monocytes are inflammatory cells recruited in large numbers to the injured heart after MI and then differentiate into macrophages at the injured site. Such engineered EVs exhibit regenerative potential of stem cells and the targeting capacity like monocytes to the injured area. As shown in a model of myocardial ischemia‐reperfusion injury, Mon‐Exos are capable of promoting angiogenesis during angiogenesis and modulating macrophage subpopulations after MI/RI, thereby regulating the progression of the disease. (Figure 9b,c).^[^ ^127^ ^]^ Besides, Gong et al. leveraged genetic engineered approach to improve the therapeutic effect of EV. They used the stromal‐derived factor 1 (SDF1a) plasmid to upregulate exosomal SDF1. SDF1a can inhibit autophagy and achieve cardiac repair function. When applied the genetically engineered EVs in vivo, SDF1‐overexpressing EV significantly inhibited autophagy in ischemic cardiomyocytes, efficiently promoting cardiac endothelial microvascular regeneration.^[^ ^128^ ^]^

##### Spinal Cord Injury (SCI)

**Figure 10:**
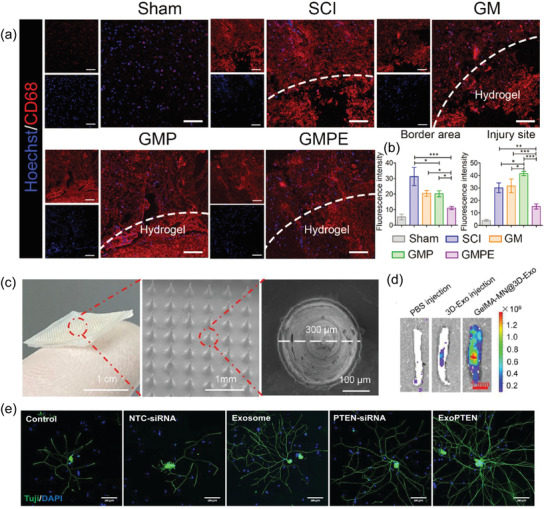
The therapeutic effect of MSC‐derived EVs on SCI. a) Fluorescence imaging of CD68‐positive cells at the injury site. b) Quantification of the intensity of CD68 fluorescence signal.Reproduced under the terms of the Creative Commons CC‐BY license.^[^ ^130^ ^]^ Copyright 2022, The Authors. Published by Wiley‐VCH. c) The morphology characterization of GelMA‐MN@Exo. d) Fluorescence imaging of the rat spinal cord tissue after transplantation of GelMA‐MN@3D‐Exo. Reproduced with permission.^[^ ^131^ ^]^ Copyright 2022, American Chemical Society. e) In vitro, ExoPTEN can promote robust axonal outgrowth of DRG neurons. Reproduced with permission.^[^ ^132^ ^]^ Copyright 2019, American Chemical Society.

EV‐derived from MSCs leads to spinal cord repair primarily through immunomodulation of the inflammatory environment and regeneration promotion.^[^ ^129^ ^]^ A recent study has used such anti‐inflammatory capacity of EVs to address the problem produced by repair materials, i.e., conductive hydrogels, that exacerbate inflammation after implantation in mice. The researchers encapsulated the bone marrow stem cell‐derived EVs (BMSC‐EVs) into a conductive hydrogel as a synergistic treatment for SCI, significantly inhibiting the early inflammation after SCI (**Figure** **10**a,b).^[^ ^130^ ^]^ In addition, hydrogel binding to EVs ensured a slow and sustained release of EVs during the initial phase of implantation. Traditional EV therapy for SCI is based on repeated local injections at the lesion site. Despite exhibiting above therapeutic abilities, this method can lead to secondary injuries and low therapeutic efficiency. Therefore, there is an urgent need to build a more convenient and controlled platform for delivery of EVs. In a recent study, using gelatin methacryloyl (GelMA) mixed with 3D cultured MSC‐derived EVs (GelMA‐MN@3D‐Exo), a microneedle array patch for local implantation was constructed (Figure 10c). Compared to conventional 2D culture, 3D cultured MSCs maintain their stemness, thus greatly improving the therapeutic efficacy of their secretory EVs (3D‐Exo) (Figure 10d), i.e., the secreted 3D‐Exo contains more active proteins and miRNAs involved in regulating the local microenvironment.^[^ ^131^ ^]^ In another study, a new EV delivery approach was developed. Guo et al. designed MSC‐derived EVs loaded with phosphatase and Tensin Homolog siRNA (ExoPTEN). After administered intranasally, the ExoPTEN significantly induced functional recovery in rats with complete SCI (Figure 10e), showing the potential of intranasal ExoPTEN to be used clinically to promote recovery in individuals with SCI.^[^ ^132^ ^]^

#### 5.1.2 Tumor Cell‐Derived EVs

It has been demonstrated that tumor cell‐derived EVs are much more likely to interact with parent tumor cells than other cells, showing a strong homing ability. To verify such homing behavior, Li et al. systemically inject HT1080 or Hela EVs to nude mice bearing a subcutaneous HT1080 tumor. Quantitative fluorescence analysis indicated that in the HT1080 tumor model, HT1080 EVs were preferentially localized at tumor tissues, but HeLa EVs were abundantly presented on the liver rather than localized to tumor sites.^[^ ^133^ ^]^ This suggests that EVs derived from specific tumor cells may be an effective delivery platform for precise tumor targeting. Currently, the use of immune checkpoint inhibitors such as humanized monoclonal antibodies (mAb) against PD‐1/PD‐L1 for cancer immunotherapy is in full swing. However, free mAb may have a series of problems in vivo: production of antidrug resistant antibodies (ADA); degradation by proteases; production of extra‐tumor toxicity. Attracted by EVs’ homing ability, Chen et al. opted for an EV‐based alternative. Using CRISPR/Cas9 to knock down the instinctive B2M and PD‐L1 on cancer cells, the variant human PD‐1 protein (havPD‐1) was then overexpressed on cell membrane. The secreted havPD‐1 expressing EVs had a breast tumor‐homing effect, thereby mitigating the extracellular toxicity of the target cells. Furthermore, havPD‐1 interacts with and blocks PD‐L1 on target cells efficiently activating cytotoxic T cells.^[^ ^134^ ^]^ Despite generating promising therapeutic outcomes, the use of tumor cell‐derived EVs in cancer therapy should be approached with caution, as they may potentially drive tumor progression and metastasis. A recent study might provide insight into the future study into tumor cell‐derived EV. Sepsis is a disease caused by immune hyperactivation and cytokine storm. Epidemiological investigations have shown that melanoma patients experience significantly lower rates of sepsis than the general population. Additionally, after sepsis induction, experimental tumor mice have a high survival rate. These all imply that tumor may be associated with the suppression of sepsis‐related immune hyperactivation. Inspired by this assumption, Li et al. used lipopolysaccharide (LPS) to stimulate tumor cells cultured in vitro and found that the induced EVs were more protective against sepsis compared to uninduced ones. Further analysis revealed that the induced tumor‐derived EVs exerted their protective effects mainly through seven key miRNAs. Using the aforementioned microRNAs, a biomimetic immunosuppressive EV has been designed that provides significant protection against sepsis in vivo. Such bionic EVs can both leverage tumor immunosuppressive properties while avoiding the potential risks of tumor cell‐derived EVs. Additionally, this opens up new avenues for the study of therapy for sepsis and cytokine‐related disease.^[^ ^135^ ^]^

#### 5.1.3 Macrophage‐Derived EVs

Tumor‐associated macrophages can be divided into two subtypes, anti‐tumor M1 macrophages that dominate the antitumor microenvironment, and pro‐tumor M2 macrophages that dominate the pro‐tumor microenvironment.^[^ ^136^ ^]^ While M1 macrophages generate pro‐inflammatory cytokines, M2 macrophages produce anti‐inflammatory cytokines.^[^ ^137^ ^]^ Interestingly, the two phenotypes can be switched, demonstrating the extreme plasticity of macrophages. M1 macrophage‐derived EVs inherit the pro‐inflammatory capacity of M1 macrophages. Appropriately engineered EVs have potential as immune enhancers for cancer therapy due to their high targeting and efficient pro‐inflammatory capacity.^[^ ^138^, ^139^ ^]^ For instance, Gunassekaran et al. engineered M1 macrophage‐derived EVs to enable EVs can both target tumor‐associated macrophages (TAMs) and reprogram TAMs to the M1 type. To target the interleukin‐4 receptor (IL4R) in TAMs, an IL4R‐binding peptide was first conjugated on EVs surface. To enhance the polarization to M1 type, they transfected M1 type EV with NF‐*κ*B p50 siRNA and miR‐511‐3p (named IL4R‐Exo(si/mi)). The researchers reported that systemic administration of IL4R‐Exo (si/mi) significantly downregulated target genes in M2 macrophages, decreased M2 cytokines, and increased M1 cytokines thereby inhibiting tumor growth.^[^ ^140^ ^]^

M2 macrophage‐derived EVs inherit the characteristics of M2 macrophages, with homing properties and anti‐inflammatory ability. Using M2 macrophage‐derived EV to deliver cargoes, such as microRNAs (miRNAs),^[^ ^141^ ^]^ interleukin‐10 (IL‐10)^[^ ^131^ ^]^ can effectively promote the polarization of macrophages to M2 type, thereby enhancing treatment efficiency. For instance, Liu et al. developed a method of producing EV (IL‐10^+^ EV) loaded with interleukin‐10 (IL‐10) by engineered macrophages, and investigated the therapeutic effect of IL‐10^+^ EV in mice with ischemic acute renal injury. As a result of the integrins on EV surface, IL‐10 delivered through EV enhances its stability, as well as its ability to target kidney. Additionally, IL‐10+ EV effectively targets macrophages in the tubulointerstitial space, triggering them to polarize in the presence of IL‐10+ EV. As a result of the treatment with IL‐10+ EV, the damage to renal tubular cells and inflammation caused by ischemia/reperfusion injury was significantly reduced, and chronic kidney disease was effectively prevented.^[^ ^142^ ^]^

#### 5.1.4 Platelet‐Derived EVs

Platelets are important cells not only due to their role in hemostasis, but also their active involvement in inflammation development.^[^ ^143^ ^]^ Evidences have shown that platelet‐derived EVs are able to bind to a wide range of receptors on the activated and inflamed vascular walls through a series of mechanisms, indicating their intrinsic affinity with inflammatory sites.^[^ ^144^ ^]^ Ma et al. engineered platelet‐derived extracellular vesicles (PEVs) to deliver anti‐inflammatory drug [5‐(p‐fluorophenyl)‐2‐ureido]thiophene‐3‐carboxamide (TPCA‐1). The PEVs accumulated well at the site of pneumonia. According to the results of the model of acute lung injury (ALI) mice, TPCA‐1‐PEVs inhibited the infiltration of pulmonary inflammatory cells and milded a local cytokine storm of inflammatory cells. According to studies, PEVs can target different inflammatory sites selectively, implying that they can serve as a powerful and flexible platform for targeting inflammation on a wide variety of organs and tissues.^[^ ^145^ ^]^ Nowadays, MSC‐derived EVs are the most commonly exploited EV therapy. Platelet‐derived EVs received comparatively less attention. However, to generate clinically relevant EV doses, MSCs require a stage of isolation and in vitro expansion. Unlike the former, PEVs can be generated directly from collected platelet concentrates. This suggests a clear clinical advantage of platelets originated EVs as the derivation of platelets.^[^ ^146^ ^]^

### 5.2 Engineered EVs as Drug Delivery System for Cancer Therapy

Viruses and synthetic vectors have been successfully used as conventional drug delivery devices to encapsulate various drugs. However, both of these systems come with drawbacks of their own. Exogenous vectors, such as viruses, may induce undesired immune responses, leading to toxicity and therapeutic failure. Furthermore, the amount of drug loaded into the viral vector is similarly limited. Likewise, synthetic vectors, such as surface‐modified liposomes, can provide relative cell‐type specificity. Still, the liver toxicity and rapid clearance of liposome delivery systems pose great challenges for clinical applications.^[^ ^147^ ^]^ Compared with the above vectors, natural sources of EV offer the advantage of avoiding phagocytosis, prolonging the half‐life of therapeutic agents, and reducing immunogenicity.^[^ ^148^ ^]^ Of note, EV can efficiently penetrate the blood–brain barrier, providing new insights and directions for treating brain tumors such as gliomas. Furthermore, EVs can be readily engineered by different approaches. EVs have shown to be more efficient, targetable and therapeutically effective when they are engineered.^[^ ^149^ ^]^ Engineered EVs are, therefore potential drug carrier that can be used in various situations from chemotherapy to gene therapy.

#### 5.2.1 Chemotherapy

Various chemotherapeutic drugs can be loaded in EVs, including plant‐derived natural compounds, such as triptolide (TPL), and synthetic drugs, such as paclitaxel.^[^ ^150^, ^151^ ^]^ To enhance the tumor target capability of drug‐loaded EVs, Gu et al. bound tumor necrosis factor‐related apoptosis‐inducing ligand (TRAIL) to the EV surface. In vitro, TRAIL‐engineered exosomes for loading TPL (TRAIL‐Exo/TPL) efficiently binds to the pro‐apoptotic receptor DR5, activating both the extrinsic TRAIL pathway and the intrinsic mitochondrial pathway to induce apoptosis. In a melanoma nude mouse model, triptolide encapsulated in TRAIL‐Exo/TPL significantly inhibited tumor development and reduced the toxicity of TPL. This work provides a promising EV‐based drug delivery system for targeted melanoma therapy.^[^ ^152^ ^]^ Although the above small molecule drugs are effective for treating the primary tumor, they show limited effectiveness in inhibiting tumor metastasis due to inefficient elimination of circulating tumor cells (CTCs). To achieve such a dual effect, Shen et al. encapsulated reactive oxygen species (ROS)‐sensitive mono‐ thioether‐linked paclitaxel‐linoleic acid prodrug (PTX‐S‐LA) and cucurbitacin B into polyethylene glycol‐block‐poly(ε‐caprolactone) polymer to prepare double drug‐loaded micelles, which was then loaded into EVs derived from tumor cells to achieve sequential activation of a biomimetic prodrug nanoplatform in an exocrine‐like manner. This design effectively targets the primary tumor and specifically traps and removes CTCs. After cell uptake, the EV‐based drug delivery system release cucurbitacin B, which can significantly block the adhesion, migration, and invasion of cancer cells and inhibit tumor metastasis through the regulation of FAK/MMP signal pathway. At the same time, cucurbitacin B‐induced ROS production stimulates PTX‐S‐LA prodrug activation, killing two birds with one stone.^[^ ^153^ ^]^ Anticancer drugs in clinical trials, such as panobinostat, have broad activity against many cancer types, but high doses often cause systemic toxicity, such as diarrhea and cardiac arrhythmias. To improve their clinical tolerability and antitumor effectiveness, it has been suggested that these epigenetically targeted drugs could be selectively delivered and activated locally at tumor sites through the action of EVs. In a recent study, Santamaría et al. employed tumor‐derived angiogenesis EVs to supply palladium catalysts to cancer cells, enabling them to activate chemotherapy drugs in situ through palladium catalysts. Due to the targetability of EVs, the EVs prefer to transfer the catalyst to the primordial cells. Once inside the cells, the catalyst converts inactive anticancer drugs into active and toxic forms, thereby selectively killing tumor cells.^[^ ^154^ ^]^

#### 5.2.2 Gene Therapy

A rapid growth in the development of CRISPR/Cas9 systems provides attractive prospects for efficient, targeted genome editing therapeutic strategies for cancer. Previous studies have demonstrated that viral vectors and liposomes for CRISPR/Cas9 system delivery risk raise off‐target mutagenesis and immunogenicity.^[^ ^155^ ^]^ A good biosafety profile and targeting potential make EV an attractive option for CRISPR/Cas9 delivery. As reported by Kim et al., cancer‐derived EVs can function as effective natural carriers for the delivery of CRISPR/Cas9 plasmids. By selectively accumulating in ovarian cancer tumors, cancer‐derived EVs provide effective in vivo delivery compared with epithelial cell EVs. Further, CRISPR/Cas9‐loaded EVs were found to be potent in suppressing poly (ADP‐ribose) polymerase‐1 (PARP‐1) expression, ultimately causing apoptosis in ovarian cancer cells. Additionally, inhibition of PARP‐1 by CRISPR/Cas9 technology increases the chemosensitivity to cisplatin, resulting in synergistic cytotoxicity.^[^ ^156^ ^]^ The results of these studies suggest that cancer‐derived EVs may be very promising future therapeutic vehicles for CRISPR. Besides, EVs can also help to deliver CRISPR ribonucleoproteins (RNPs). Majeau et al. load RNPs into serum EVs, which consist of SpCas9 proteins and guide RNAs (gRNAs), revealing that EVs are effective transporters of RNPs in vitro. In vivo, the expression of tdTomato fluorescent protein was significantly restored in muscle fibers of Ai9 mice.^[^ ^157^ ^]^ The well‐designed plasmid has become a widely used CRISPR tool due to its cheapness, ease of use, and the ability to incorporate marker genes (e.g., antibiotic genes). Nevertheless, plasmids can stay in the host cell for weeks. During this period, all or part of the sequence of plasmid DNA may be randomly integrated into the host genome, leading to nontarget gene editing. RNPs, complexes of Cas9 proteins and guide RNAs, rapidly cut the target sequence after delivery and are quickly degraded after that, thereby avoiding cutting the wrong site.^[^ ^158^ ^]^ In addition, once the host belongs to a sensitive cell type (e.g., pluripotent stem cells), the presence of exogenous DNA may trigger the activation of cyclized GMP‐AMP synthase (“accelerator” of immune system),^[^ ^159^ ^]^ resulting in cellular toxicity. The current challenge of applying EVs to deliver Cas9/sgRNA RNP complex is how to encapsulate Cas9 into EVs effectively. Existing EV‐based methods for RNPs encapsulation, e.g., chemically‐induced dimerization strategy, are relatively complicated to operate. Therefore, there is an urgent need for a simple yet effective encapsulation method to enable the delivery of RNPs. Kim et al. developed a straightforward method. They attached the myristoyl‐group to the N‐terminus of Cas9 covalently, serving as an anchor for Cas9 to associate with the cell membrane, effectively encapsulating CRISPR/Cas9 into EVs.^[^ ^160^ ^]^ Still, more EV‐based methods for RNPs encapsulation should be developed in the future. In addition to the gene editing system CRISPR/Cas9, considerable amounts of studies have been devoted to the investigation of the function of miRNAs carried by EVs for seeking their potential application in gene therapy. For example, miR‐466 was highly expressed in adipose‐derived MSC (Ad‐MSC) EVs and targeted mRNA coding for toll interleukin 1 receptor domain containing adaptor protein (TIRAP), leading to *TIRAP* gene inhibition. Further, Shi et al. demonstrated the immunomodulatory effects of miR‐466 in a pneumonia model.^[^ ^161^ ^]^

#### 5.2.3 Immunotherapy

**Figure 11:**
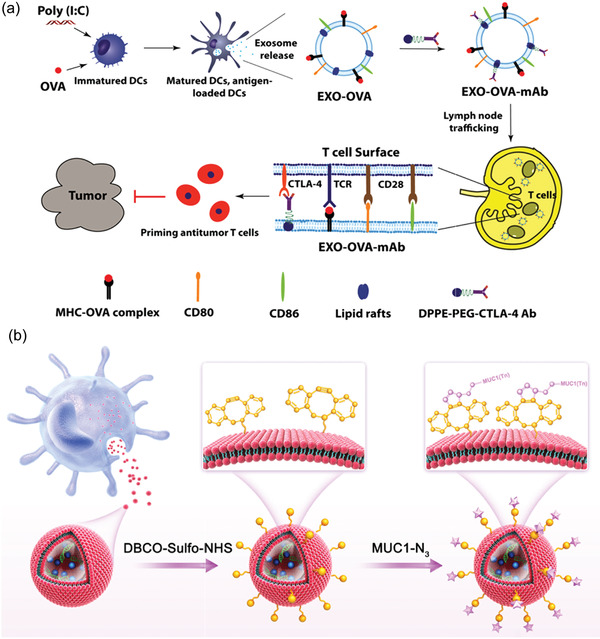
Engineered EVs for immunotherapy. a) Schematic representation of EXO‐OVA‐mAb targeting lymph nodes and enhancing tumor‐specific T cell responses. Reproduced with permission.^[^ ^163^ ^]^ Copyright 2020, Elsevier. ILN: inguinal lymph nodes. b) Schematic diagram of the construction of MUC1‐Dex. Reproduced with permission.^[^ ^164^ ^]^ Copyright 2021, Elsevier.

Recent years have seen an increase in the number of cancer vaccines that induce specific antitumor immune responses via the delivery of tumor‐associated antigens to dendritic cells (DCs) in lymphoid organs as well as appropriate adjuvants. In clinic, however, there are “protective shields” such as immune checkpoint components (e.g., negative immunomodulators such as cytotoxic T‐lymphocyte antigen 4 (CTLA‐4)) that result in ineffective delivery of TAAs and adjuvants.^[^ ^162^ ^]^ Therefore, more effective strategies are needed to developed to enhance immune response. Recently, engineered EVs activated tumor‐specific T cell immune responses have emerged as a promising alternative strategy for cancer vaccination. Phung et al. used EVs to load anti‐CTLA‐4 antibodies (EXO‐OVA‐mAb), which were prepared from activated dendritic cells pulsed with ovalbumin (**Figure** **11**a). In vitro, EXO‐OVA‐mAb significantly activated T cells. In vivo, EXO‐OVA‐mAb accumulated in large amounts in lymph nodes. With this innovative approach, immune checkpoint inhibition, and cancer vaccination are more effective against tumors.^[^ ^163^ ^]^

Many cancers, such as ovarian, prostate, breast, and rectal, overexpress the transmembrane glycoprotein MUC1. As a result of aberrant glycosylation, the amino acid residues of MUC1 become tumor‐associated carbohydrate antigen, which can also be used as tumor‐specific antigen for immune enhancement. Using dendritic cell‐derived EV (Dex) as a delivery system and adjuvant, Zhu et al. designed a highly efficient MUC1 glycopeptide‐Dex conjugate (MUC1‐Dex) (Figure 11b). Their results showed MUC1‐Dex enhanced T cell cytotoxicity as well as IgG antibody titers. Furthermore, MUC1‐Dex significantly inhibited tumor growth and prolonged survival in the mice model.^[^ ^164^ ^]^

## 6 Conclusions and Perspectives

Research on the application of EVs in theranostics has been intensive in the past. However, there are still some obstacles to overcome before employing EVs as a successful theranostic platform. First of all, controlling the purity of EVs, especially considering the size overlap between exosomes and other extracellular vesicles (e.g., microvesicles), is of primary importance. Traditional EVs purification methods have disadvantages, such as low purity and long operation time, making it difficult to meet the requirements of research and clinic. Therefore, it is crucial to develop more superior EV purification strategies.

Another challenge that needs to be overcome in using EVs as treatments is the insufficient efficacy of natural EV therapies. Previous studies revealed that such accidents were primarily due to their off‐target properties. But, as we summarized in this review, engineering strategies can solve this problem by introducing desired molecules onto the surface of EVs with chemical modification, genetic engineering or by fusing EV with liposome structures. Despite the expected results, there are still some challenges in the engineering of EVs due to the immaturity of the technology: a) Developing chemical modifications that enhance EV targeting while maintaining membrane integrity; b) which cells are suitable as parent cells for engineered EVs; c) most of the current experiments are carried out in vitro, but ultimately to be applied to the clinic, how to ensure the same effect in vitro and in vivo. Future research is suggested to answer these questions.

EVs reflect the original state of their parent cells. In recent years, the development of detecting EV has gradually accelerated. High‐throughput technology for resolving practical clinical problems is continually being explored. With the advances in biosensors, it has been possible to improve detection performance using cross‐technology, expand the platform based on integrated functions, and push the use of new biosensors in clinical practice in an effort to improve detection performance. The related research can be divided into the following specific contents: a) Fundamental theory of biosensors based on specific biomolecules; b) The internal logic of converting biological signals into electrical signals in the integrated biosensor; c) Adaptors or probes customized to enrich EVs and improve the quality of sensing signals. Specifically, in the process of rapid iteration, the direction of technical optimization is convergence and integration. At present, several suitable detection methods have been developed in the fields of optics, electrochemistry, electromagnetism, and so on. In the future, the combination of technologies in different fields will be closer. The miniaturization, automation, and convenience of microfluidic technology also show strong applicability in EVs detection, which has the potential for high‐throughput and rapid separation and detection. However, with the increasing understanding of the characteristics of EVs, many complex detection methods have emerged. The numerous and complex technical means make researchers unable to reach a common consensus. How to balance the sensitivity, specificity, time efficiency and economic cost of EVs is still a problem worth solving in the future. In the process of practical application, biosensors composed of different biomaterials and electronic materials may become one of the promising ways, which will promote the development of portable EV detection as much as possible and also feeds the researchers' further understanding of EVs, making it complementary to promote the application process of EV detection.

Engineered EVs have shown promising therapeutic effects in disease treatment. However, choosing an appropriate cell source for EV production according to the therapeutic application is very important. Existing problems are as follows: a) while MSC‐derived EV exhibits a strong tissue repair capacity, it also potentially promotes tumor growth during cancer treatment, which requires further studies. b) Tumor cell‐derived EV has a strong tumor homing effect, yet their role in tumor progression has been described as metastatic. Thus, future studies may focus on identifying substances, such as integrins or lipids, that play a key role in the EV homing mechanism or choose to construct bionic EVs to address potential insecurities. Another focus will be to identify the types of cellular sources of EV that can accumulate highly in tumor tissues, c) to generate clinically relevant EV doses, MSCs require a stage of isolation and in vitro expansion; unlike the former, PEVs can be generated directly from collected platelet concentrates. Furthermore, platelets are non‐nuclear, mitigating safety concerns related to possible teratogenic risks. These all suggest that further research concentrating on PEV is worthwhile in the future.

Overall, advanced nanotechnology provides extensive possibilities for EVs theranostics. We believe these will further optimize their clinical translation as a diagnostic and therapeutic platforms in the near future.

## Conflict of Interest

The authors declare no conflict of interest.
